# Physical fighting, fighting-related injuries and family affluence among Canadian youth

**DOI:** 10.1186/s12889-016-2886-3

**Published:** 2016-02-29

**Authors:** Maya Djerboua, Bingshu E. Chen, Colleen M. Davison

**Affiliations:** Department of Public Health Sciences, Queen’s University, 62 Fifth Field Company Lane, Kingston, ON K7L 3 N6 Canada; Clinical Research Centre, Kingston General Hospital, Kingston, ON Canada; Department of Emergency Medicine, Queen’s University, Kingston, ON Canada

**Keywords:** Adolescence, Epidemiology, Injury, Physical fighting, Family affluence

## Abstract

**Background:**

Physical fighting is an assaultive behaviour that can lead to injury. Family affluence is a health determinant that can influence injury. This study examines the relationship between family affluence and two outcomes: physical fighting and fighting-related injury in Canadian adolescents. Three measurements were used to represent family affluence and assess whether these measures demonstrated different associations with these outcomes.

**Methods:**

Canadian data from the 2009/2010 Health Behaviour in School-aged Children Study were used. It consists of a nationally representative sample of 26,078 grade 6–10 students. A subset analysis of 10,429 grade 9–10 students was conducted to account for additional confounders. Modified Poisson regression was used to compare the risk of physical fighting and fighting-related injury in youth from different levels of family affluence. Three indicators were used to represent family affluence: self-perceived affluence, a family affluence scale (FAS), and area-level average household income.

**Results:**

The overall prevalence was 35.6 % for physical fighting and 2.7 % for fighting-related injuries. Both outcomes were more frequent in males than females. An inverse gradient was present where risk for both outcomes increased with decreasing levels of affluence irrespective of the affluence measurement. The self-perceived affluence variable showed a significantly stronger gradient in girls than boys for both outcomes. For both outcomes, FAS showed a similar inverse gradient within females, but a threshold effect in males where there was a strong effect in the low FAS group, but a null effect in the moderate FAS group. The area-level income variable presented a significantly higher likelihood for physical fighting only in females (*p* = 0.001–0.075). For fighting-related injury, none of the area-level income models showed significant risk estimates with the exception of the bivariate association where low income females were twice as likely to report a fighting-related injury compared to higher income groups (*p* = 0.030). Post hoc power calculations indicate that there was not sufficient power to detect injury effects associated with the area level income measure.

**Conclusion:**

It appears that a socioeconomic gradient exists where lower affluence is associated with a higher risk of reporting a physical fight and fighting-related injury irrespective of the measure used. While the patterns were generally the same with all three measurements, the strength of this gradient varied across measures. This demonstrates that each indicator may measure different aspects of affluence. Further analyses are needed to explore concepts and mechanisms underlying each affluence measure.

## Background

One common manifestation of violence is physical fighting, which is an assaultive behaviour that is a significant public health issue among young people worldwide. Fighting is a concerning behaviour since it has been proposed as one of the earliest markers for multiple risk behaviours such as substance use, truancy, and other problem behaviours [1], and is consistently shown to cause injury [1]. Injury is one of the most important negative health outcomes seen in young people today, and physical fighting is one of the most common causes of serious injury requiring medical attention in young people [2]. According to the World Health Organization (WHO), injuries resulted in tens of millions of young people under the age of 18 requiring hospital care and 950 000 deaths each year worldwide [3]. Adolescent injuries are a significant concern due to their enormous burden on adolescents, families and communities, with costs associated with premature death, pain, disability, reduced productivity, and emotional trauma [2].

There are numerous factors related to fighting and injuries. One important factor is family affluence or wealth. Previous studies have examined the association between wealth and one’s predisposition for violence [4]. A recent study conducted using data from 79 countries further found that country wealth was a robust determinant of youth violence such as physical fighting and bullying, where increases in per capita income corresponded with less physical fighting and bullying [5]. There is a general scarcity of literature in Canada regarding physical fighting and injuries specific to fighting among young people and its relationship with family affluence. Another international study conducted in 30 countries, including Canada, found that higher absolute wealth is associated with a lower likelihood of frequent fight involvement [4]. Previous research has also assessed the association between measures of wealth and its related construct, socioeconomic status (SES), on adolescent injuries. The results reporting the relationship between affluence and adolescent injuries were unclear though and authors noted that there was no optimal measurement for family affluence [6]. Furthermore, the results for these relationships varied by injury cause, type and severity. For example, higher SES was associated with a higher risk of sports-related injuries and lower SES was associated with a higher likelihood of fighting-related injuries [7]. There is also the possibility that medically treated injury events may be over-reported in more affluent schools due to greater access to healthcare resources compared [6]. In order to have a more thorough understanding of these associations, there is a need for research on multiple indicators of family affluence and studies that include context and cause-specific injury information [8].

The current study examines the relationship between family affluence and two outcomes: physical fighting and fighting-related injury in Canadian adolescents. Three different measurements were used to represent family affluence to further assess whether these measures demonstrated different associations with physical fighting and fighting-related injury.

## Methods

### Data source

This study used Canadian data from the Health Behaviour in School-aged Children (HBSC) study. It is a cross-sectional survey that was developed in collaboration with the WHO with the intent of studying health determinants and behaviours in young people 11–15 years of age [9]. The HBSC study protocol and this specific secondary analysis received ethics approval from the Queen’s University General Research Ethics Board (File #: 6011541).

### Study sample

A two-stage cluster sampling approach was employed for the most recent 2009/2010 HBSC cycle where students were clustered within schools. Consent was initially obtained (in order) by the school jurisdictions, school principals, and parents. After each level of consent is achieved, participation from students is voluntary. HBSC surveys were then administered to classrooms during 45–70 min sessions to collect data. A response rate of 77 % out of the eligible participants was recorded for the most recent 2009/2010 HBSC study. This resulted in an original sample size of 26,078 students from 436 schools in 11 provinces and territories. Another analysis with only grade 9–10 students was undertaken to consider potential covariates that were not available in the grade 6–8 version of the HBSC survey, such as those pertaining to drug use (as these were not asked of younger students). This resulted in a sub-sample size of 10,429 grade 9–10 students.

### Main exposure: family affluence

Family affluence is the main exposure of this study. Many variables were available to represent this construct, and three methods of measuring family affluence were used for data analysis: self-perceived affluence, a family affluence scale (FAS), and area-level average household income.

*Self-perceived family affluence* was indicated by a question in the student survey that asked students the following question: ‘How well off do you think your family is?’ These responses were represented as a five-point scale: ‘very well off’, ‘quite well off’, ‘average’, ‘not very well off’, and ‘not at all well off’. Responses were re-categorized as three categories for the analysis: high (‘very well off’, ‘quite well off’), moderate (‘average’), and low (‘not very well off’, ‘not well off at all’).

The second method used for measuring family affluence was the *Family Affluence Scale II (FAS)*, which is a validated measure of four questions that uses a set of material items to reflect family expenditure where possession of greater numbers of these items can represent increasing affluence, or lacking them can represent material deprivation [10]. It is useful since students may not have an accurate idea of how much money their guardians make or have, and the FAS is an alternative approach that approximates affluence based on the kinds and quantity of items the student’s family can afford. Items in the FAS scale include: 1) having a bedroom for oneself (‘Do you have your own bedroom for yourself?’), 2) number of vehicles (‘Does your family own a car, van or truck?’), 3) family vacations in the past 12 months (‘During the past 12 months, how many times did you travel away on holiday (vacation) with your family?’), and 4) number of computers (‘How many computers does your family own?’). Each question was worth up to 2 points (3 for family vacation question). Responses from all four FAS questions were totaled to create a FAS score which ranged from 0 to 9. For this study, the FAS score was divided into 3 ordinal categories to represent an individual’s family affluence: low affluence (0–2), moderate affluence (3–5), and high affluence (6–9). This categorization is based on recommendations from previous studies [11, 12].

*Area-level income* was the third method for measuring family affluence. The postal code of the school that each student attended was available in the HBSC data. The school postal code was linked and merged with information on the average household income among private households within a 1 km buffer of the school from the 2006 Statistics Canada Census Subdivision data. Average income was calculated by dividing the aggregate income of the group of families or households within this 1 km school buffer by the number of families or households in that respective group. A private household is a person or group of persons who occupy a private dwelling and do not have a usual place of residence elsewhere in Canada. Because of the log-normal distribution of the variable, the area-level average household income measurement was divided into percentile-based tertiles.

All three measurements rely on different methods to quantify the concept of family affluence in adolescence. Self-perceived affluence is the most subjective measure since it relies on self-report to measure an adolescent’s affluence, and depending on what their frame of reference or definition of “well off” is, it may be variable. FAS is a more objective measurement in that it aims to use material items to measure family expenditure. FAS also relies on HBSC survey questions to gauge material wealth, however it does indirectly measure wealth without asking an adolescent about their parent’s income. This is done primarily to decrease the likelihood of non-responses. Area-level income is a more objective affluence measurement again since it relies on income Census data reported directly by parents. Despite these different approaches to measuring family wealth, these measurements are expected to be correlated and yield similar results.

### Outcome 1: physical fighting

Physical fighting was assessed with the question ‘During the past 12 months, how many times were you in a physical fight?’ Five ordinal responses were available, ranging from ‘none’ to ‘4 times’. These responses were re-categorized as a dichotomous response for analysis: ‘none’ and ‘yes (one or more times)’.

### Outcome 2: fighting-related injury

Fighting-related injury was assessed using two survey items. The first question asked the participant ‘During the past 12 months, how many times were you injured and had to be treated by a doctor of nurse?’ The second question ‘What were you doing when this one most serious injury happened?’ was asked to assess what the cause of the participants’ one most serious injury was. If participants selected ‘Yes’ in response to whether they were injured in the past 12 months and selected ‘Fighting’ as the cause of their one most serious injury, then they were coded as having a fighting-related injury. Respondents who either were not injured in the past 12 months or were injured by other means besides fighting were coded as not having a fighting-related injury.

### Potential covariates

Potential covariates were identified based on previous literature and were adjusted for in the analysis. Confounders were selected based on either their association with the outcomes of physical fighting and fighting-based injuries, or their independent affiliation with both family affluence and the outcomes without being on the causal pathway. Effect modification was assessed and determined to be significant based on the interaction term (between each family affluence variable and sex) in the regression models while adjusting for other factors.

Demographic factors such as sex and age were previously established to be important predictors for physical fighting participation and injury and thus were considered a priori as covariates [13–19]. Other potential confounders that were assessed in the analysis were academic performance [20], happy home life or supportive families [21–23], respectful school environment (school connectedness) [13, 24], caring and understanding teachers [22, 25], extracurricular activities [22], sports involvement [22], and drug and alcohol use [26–28]. Drug use questions were only available for grade 9–10 students, therefore this variable was only considered for the analysis of grade 9–10 participants.

### Survey weights

The HBSC data were weighted within grades by province or territory to ensure that the results were proportionate and nationally representative of the actual student population. Grade groups that were over-represented in provinces and territories were given a weight of <1, and those who were under-represented were given weights of >1. The survey weights for each grade ranged from 0.017–3.655.

### Statistical analysis

The association between family affluence and the outcomes of physical fighting and fighting-related injury was assessed using modified Poisson regression analyses with log link function to estimate relative risks (RR) and 95 % confidence intervals (CI). All statistical analysis procedures were conducted using the PROC GENMOD procedure from SAS Version 9.4 software (SAS Institute Inc., Cary, North Carolina). The analysis took into consideration the clustered nature of the data where students (individual-level) were nested within schools (area-level). This was done by using generalized estimating equations (GEEs) to create robust error estimates [29]. The highest affluence category was chosen as the reference group for each of the multi-level analyses. A two-stage approach was undertaken for the analysis. Firstly, bivariate models were fitted for each affluence exposure and outcome. Secondly, multivariate regression models were fit while stratifying by sex and adjusting for confounders that were chosen based on a backwards elimination criteria of *p* < 0.15 to create the most parsimonious model.

## Results

The individual- and area-level characteristics of the 2009/2010 HBSC sample can be seen in Table 1. Sex was a significant effect modifier for all physical fighting outcome models based on type 3 test statistics (p_interaction_ < 0.05). Therefore results were stratified by sex.

**Table 1:** Description of physical fighting and fighting-related injuries by individual and area-level affluence characteristics in the 2009/2010 HBSC study

		Physical fighting						Fighting-related injury					
		Overall		Males		Female		Overall		Males		Females	
	Overall N	n	(%)	n	(%)	n	(%)	n	(%)	n	(%)	n	(%)
Overall	26078	8945	(35.6)	5944	(48.7)	2997	(23.2)	665	(2.7)	443	(3.7)	222	(1.7)
*Individual-level characteristics*													
Self-perceived affluence													
Low	2278	964	(42.3)	555	(54.3)	408	(32.6)	104	(4.6)	51	(5.0)	53	(4.2)
Moderate	8103	3073	(37.9)	1882	(51.0)	1190	(26.9)	267	(3.3)	168	(4.6)	98	(2.3)
High	13746	4504	(32.8)	3228	(46.5)	1274	(18.7)	268	(1.9)	202	(2.9)	67	(1.0)
Total	24127	8541		5666		2873		639		421		218	
Family affluence scale													
Low	576	253	(43.9)	147	(54.9)	106	(34.3)	41	(7.2)	33	(12.7)	8	(2.5)
Moderate	7734	2688	(34.8)	1687	(46.1)	999	(24.5)	193	(2.5)	115	(3.2)	78	(1.9)
High	15295	5338	(34.9)	3607	(49.2)	1731	(21.7)	376	(2.5)	255	(3.5)	121	(1.5)
Total	23605	8279		5441		2836		610		403		206	
*Area-level characteristics*													
Average household income													
Low	8251	3006	(36.4)	1910	(48.2)	1096	(25.6)	230	(2.8)	138	(3.6)	92	(2.2)
Moderate	8178	2931	(35.8)	1930	(49.9)	999	(23.2)	224	(2.8)	147	(3.8)	77	(1.8)
High	8415	2872	(34.1)	2010	(47.7)	862	(20.5)	197	(2.4)	147	(3.5)	50	(1.2)
Total	24884	8809		5850				651		432		219	

The overall prevalence was 35.6 % for physical fighting and 2.7 % for fighting-related injuries. Both outcomes were more frequent in males than females. However, the relationship between family affluence and both outcomes varied depending on the affluence measurement that was used in each model. For the self-perceived affluence variable, the prevalence of physical fighting was highest in the low affluence group and the prevalence decreased with each increasing affluence category (low: 42.3 %, moderate: 37.9 %, high: 32.8 %). This pattern was also observed for the FAS (low: 43.9 %, moderate: 34.8 %, high: 34.9 %) and the area-level income measurement (low: 36.4 %, moderate: 35.8 %, high: 34.1 %).

Fighting-related injury also presented an inverse gradient where the prevalence was 4.6, 3.3 and 1.9 % for the low, moderate and high self-perceived affluence groups respectively. The FAS and area-level income variables showed a slight gradient effect although some of the affluence categories contained the same prevalence estimates: (low: 7.2 %, moderate: 2.5 %, high: 2.5 % for FAS; low: 2.8 %, moderate: 2.8 %, high: 2.4 % for area-level income).

Table 2 shows the results of the regression analyses for the physical fighting outcome. The bivariate analysis for the self-perceived affluence measure showed that low affluence males had a 14 % higher risk of being in at least one physical fighting compared to high affluence males (95 % CI: 1.05–1.25), while the risk increase was 8 % for moderate affluence males (95 % CI: 1.02–1.15) as compared to high. Within the female stratum, participants with low self-perceived affluence had a 66 % increased likelihood of reporting one physical fight compared to those with high self-perceived affluence (95 % CI: 1.45–1.90) while the moderate affluence group had a 39 % higher risk (95 % CI: 1.27–1.53). When adjusted for all significant confounders, the risk for both strata decreased although the risk within females of low affluence remained significantly higher compared to the referent (RR = 1.39, 95 % CI: 1.20–1.60), and females of moderate affluence had a 30 % increased risk compared to high affluence females (95 % CI: 1.17–1.43). The relationship between affluence and physical fighting within males was no longer significant when adjusted for all significant confounders.

**Table 2:** Modified Poisson regression - association between physical fighting and individual and area-level family affluence by sex

	Bivariate model				Multivariate model^a,b^			
	Male		Female		Male		Female	
	RR (95 % CI)	*p*-value	RR (95 % CI)	*p*-value	RR (95 % CI)	*p*-value	RR (95 % CI)	*p*-value
Self-perceived affluence								
Low	1.14 (1.05–1.25)	0.003	1.66 (1.45–1.90)	<0.001	0.99 (0.91–1.09)	0.886	1.39 (1.20–1.60)	<0.001
Moderate	1.08 (1.02–1.15)	0.005	1.39 (1.27–1.53)	<0.001	1.02 (0.95–1.09)	0.575	1.30 (1.17–1.43)	<0.001
High	1.00 (Ref)		1.00 (Ref)		1.00 (Ref)		1.00 (Ref)	
Family affluence scale								
Low	1.09 (0.92–1.28)	0.317	1.50 (1.23–1.85)	<0.001	1.01 (0.86–1.19)	0.888	1.42 (1.14–1.79)	0.002
Moderate	0.93 (0.89–0.98)	0.008	1.12 (1.02–1.23)	0.016	0.93 (0.88–0.98)	0.010	1.06 (0.97–1.17)	0.213
High	1.00 (Ref)		1.00 (Ref)		1.00 (Ref)		1.00 (Ref)	
Average household income								
Low	1.07 (0.96–1.18)	0.220	1.32 (1.15–1.53)	<0.001	1.03 (0.93–1.14)	0.536	1.26 (1.08–1.46)	0.003
Moderate	1.08 (0.99–1.17)	0.086	1.17 (1.01–1.36)	0.031	1.07 (0.98–1.16)	0.130	1.15 (0.99–1.34)	0.075
High	1.00 (Ref)		1.00 (Ref)		1.00 (Ref)		1.00 (Ref)	
Sub-cohort analysis^c^	Bivariate model				Multivariate model^b,d^			
	RR (95 % CI)	*p*-value	RR (95 % CI)	*p*-value	RR (95 % CI)	*p*-value	RR (95 % CI)	*p*-value
Self-perceived affluence								
Low	1.13 (0.98–1.30)	0.086	1.63 (1.31–2.01)	<0.001	0.95 (0.80–1.12)	0.550	1.37 (1.09–1.72)	0.008
Moderate	1.12 (1.02–1.24)	0.023	1.29 (1.11–1.51)	0.001	1.03 (0.93–1.13)	0.582	1.17 (0.99–1.39)	0.073
High	1.00 (Ref)		1.00 (Ref)		1.00 (Ref)		1.00 (Ref)	
Family affluence scale								
Low	1.11 (0.82–1.49)	0.501	1.32 (0.87–2.02)	0.194	0.94 (0.71–1.26)	0.695	1.44 (0.99–2.07)	0.052
Moderate	0.89 (0.81–0.98)	0.016	1.20 (1.02–1.40)	0.027	0.87 (0.79–0.97)	0.011	1.07 (0.89–1.27)	0.478
High	1.00 (Ref)		1.00 (Ref)		1.00 (Ref)		1.00 (Ref)	
Average household income								
Low	0.98 (0.84–1.15)	0.809	1.04 (0.82–1.30)	0.768	1.01 (0.85–1.19)	0.944	1.01 (0.81–1.25)	0.965
Moderate	0.99 (0.86–1.14)	0.917	1.03 (0.83–1.27)	0.797	1.04 (0.89–1.22)	0.581	0.97 (0.77–1.21)	0.775
High	1.00 (Ref)		1.00 (Ref)		1.00 (Ref)		1.00 (Ref)	

^a^Multivariate regression analysis adjusted for sex, age, academic performance, caring teachers, respectful students, alcohol use, happy home life, and club involvement. P(interaction) < 0.001 for self-perceived affluence, *P* = 0.016 for FAS, and *P* = 0.032 for area-level income. ^b^All multivariate models adjusted using backwards elimination with a criteria of *p* < 0.15. ^c^A sub-cohort of 10,429 grade 9–10 students was analyzed to account for confounders only available in the grade 9 and 10 version of the HBSC survey. ^d^Multivariate regression analysis for Grade 9 and 10 HBSC survey only and adjusted for sex, age, academic performance, caring teachers, respectful students, alcohol use, marijuana use, happy home life, and club involvement. P(interaction) = 0.020 for self-perceived affluence, *P* = 0.115 for FAS, and *P* = 0.723 for area-level income

The FAS variable demonstrated a similar gradient effect in the female stratum (low FAS: RR = 1.50, 95 % CI: 1.23–1.85; moderate FAS: RR = 1.12, 95 % CI: 1.02–1.23). When adjusted, the risk for each category slightly decreased but only a significant effect was detected for the low FAS category (RR = 1.42, 95 % CI: 1.14–1.79). However, the male population generated results where comparisons of the low and high FAS groups presented insignificant increases in risk, but a significant decrease in risk was present when contrasting the moderate and high FAS groups (95 % CI (bivariate model): 0.89–0.98, 95 % CI (multivariate model): 0.88–0.98).

The area-level average household income variable only presented a significantly higher likelihood for physical fighting in the female population when comparing lower income females to higher income females for the unadjusted analysis (low income: RR = 1.32, 95 % CI: 1.15–1.53; moderate income: RR = 1.17, 95 % CI: 1.01–1.36). In the adjusted analysis, lower income females were 26 % more likely to report a physical fight than high income females (95 % CI: 1.08–1.46).

Table 3 displays the regression analysis results for fighting-related injuries. Males of low self-perceived affluence were 64 % more likely to have obtained a fighting-related injury (95 % CI: 1.07–2.49) and moderate affluence males were 51 % more likely (95 % CI: 1.19–1.92) in the unadjusted models, indicating a socioeconomic gradient. However, none of the adjusted models presented a significant association between levels of self-perceived affluence and fighting-related injury within the male population. Females in general had higher risk estimates when examining the association between self-perceived affluence and fighting-related injury. When unadjusted, low affluence females had nearly 4 times the risk of obtaining a fighting-related injury compared to high affluence females (95 % CI: 2.34–6.94), and moderate affluence females had almost twice the risk (95 % CI: 1.36–3.36). After adjusting for confounders though, the risk decreased to 3 times when comparing low and high affluence groups (95 % CI: 1.69–5.11), and when examining the moderate affluence group (RR = 1.74, 95 % CI: 1.07–2.83).

**Table 3:** Modified Poisson regression- association between fighting-related injury and individual and area-level family affluence by sex

	Bivariate model				Multivariate model^a,b^			
	Male		Female		Male		Female	
	RR (95 % CI)	*p*-value	RR (95 % CI)	*p*-value	RR (95 % CI)	*p*-value	RR (95 % CI)	*p*-value
Self-perceived affluence								
Low	1.64 (1.07–2.49)	0.022	4.03 (2.34–6.94)	<0.001	1.07 (0.67–1.73)	0.775	2.94 (1.69–5.11)	<0.001
Moderate	1.51 (1.19–1.92)	0.001	2.14 (1.36–3.36)	0.001	1.32 (0.98–1.77)	0.063	1.74 (1.07–2.83)	0.024
High	1.00 (Ref)		1.00 (Ref)		1.00 (Ref)		1.00 (Ref)	
Family affluence scale								
Low	3.47 (2.05–5.87)	<0.001	1.50 (0.56–4.02)	0.424	2.10 (1.23–3.58)	0.006	1.34 (0.54–3.38)	0.528
Moderate	0.88 (0.68–1.15)	0.362	1.24 (0.90–1.70)	0.193	0.83 (0.62–1.13)	0.240	1.14 (0.81–1.60)	0.462
High	1.00 (Ref)		1.00 (Ref)		1.00 (Ref)		1.00 (Ref)	
Average household income								
Low	1.08 (0.75–1.55)	0.677	2.00 (1.07–3.73)	0.030	0.82 (0.55–1.22)	0.335	1.51 (0.77–2.97)	0.229
Moderate	1.13 (0.82–1.57)	0.450	1.66 (0.92–2.99)	0.094	1.02 (0.71–1.48)	0.903	1.62 (0.81–3.23	0.172
High	1.00 (Ref)		1.00 (Ref)		1.00 (Ref)		1.00 (Ref)	
Sub-cohort analysis^c^	Bivariate model				Multivariate model^b,d^			
	RR (95 % CI)	*p*-value	RR (95 % CI)	*p*-value	RR (95 % CI)	*p*-value	RR (95 % CI)	*p*-value
Self-perceived affluence								
Low	1.64 (0.91–2.95)	0.102	4.10 (1.77–9.53)	0.001	1.28 (0.66–2.47)	0.469	2.79 (1.12–6.91)	0.027
Moderate	1.32 (0.93–1.87)	0.126	2.25 (1.12–4.51)	0.023	1.33 (0.83–2.13)	0.233	1.76 (0.84–3.68)	0.133
High	1.00 (Ref)		1.00 (Ref)		1.00 (Ref)		1.00 (Ref)	
Family affluence scale								
Low	3.68 (1.75–7.74)	<0.001	0.78 (0.20–3.03)	0.719	1.74 (0.85–3.57)	0.129	0.80 (0.22–2.93)	0.734
Moderate	0.67 (0.45–1.01)	0.057	1.31 (0.81–2.11)	0.271	0.55 (0.32–0.94)	0.030	1.13 (0.68–1.87)	0.634
High	1.00 (Ref)		1.00 (Ref)		1.00 (Ref)		1.00 (Ref)	
Average household income								
Low	1.16 (0.68–1.98)	0.581	1.33 (0.67–2.65)	0.413	1.20 (0.72–1.99)	0.489	1.12 (0.51–2.44)	0.778
Moderate	1.42 (0.90–2.24)	0.133	1.38 (0.68–2.81)	0.377	1.61 (0.97–2.69)	0.068	1.52 (0.67–3.49)	0.319
High	1.00 (Ref)		1.00 (Ref)		1.00 (Ref)		1.00 (Ref)	

^a^Multivariate regression analysis adjusted for sex, age, respectful students, alcohol use, happy home life, and sport team. P(interaction) = 0.027 for self-perceived affluence, *P* = 0.290 for family affluence scale, and *P* = 0.172 for area-level income. ^b^All multivariate model adjusted using backwards elimination with a criteria of *p* < 0.15. ^c^A sub-cohort of 10,429 grade 9–10 students was analyzed to account for confounders only available in the grade 9 and 10 version of the HBSC survey. ^d^Multivariate regression analysis for Grade 9 and 10 HBSC survey only and adjusted for sex, age, academic performance, respectful students, alcohol use, marijuana use, and happy home life. P(interaction) = 0.385 for self-perceived affluence, *P* = 0.085 for FAS, and *P* = 0.989

When looking at the FAS variable, low FAS males were nearly 3.5 times more likely to report a fighting-related injury (95 % CI: 2.05–5.87) while moderate FAS males were 22 % insignificantly *less* likely to report a fighting-related injury when unadjusted (95 % CI: 0.68–1.15). However after adjusting for all covariates in the multivariate models, the effect estimate still demonstrated an increased risk for fighting-related for lower income males (RR = 2.10, 95 % CI: 1.23–3.58) and moderate FAS males (RR = 0.83, 95 % CI: 0.62–1.13) when compared to the referent group. Although the effect estimate for the adjusted model slightly decreased in comparison to the unadjusted model, the effect for low FAS males on injury was still significant when adjusted for covariates. For females, the association between FAS and fighting-related injury showed an inverse gradient where risk estimates increased with lower FAS groups, although no estimates were significant.

In regards to area-level income, post hoc power calculation revealed that we did not have sufficient power to detect true injury effects if they did in fact exist. None of the models showed significant risk estimates with the exception of the bivariate association within females where low income females were twice as likely to report a fighting-related injury (95 % CI: 1.07–3.73).

## Discussion

This study is unique in its contribution of assessing the relationship between family affluence and physical fighting and fighting-related injury among Canadian adolescents by using several indicators for family affluence and focusing specifically on injuries caused by fights as opposed to general injuries. Previous research has suggested that lower levels of affluence were generally associated with a higher risk of participating in a physical fight and obtaining a fighting-related injury. For this particular study, these associations varied in strength depending on the affluence measurement that was used, and within males and females.

With regard to the overall prevalence of physical fighting (35.6 %) and fighting related injuries (2.7 %) in the entire HBSC population, the findings are consistent with what is seen in previous research. The sex-based differences, highlighted for fighting and fighting-related injury prevalence, are consistent with prior findings. A unique finding in this research was that sex was a significant effect modifier that interacted with family affluence. When assessing the prevalence of physical fighting and fighting-related injury without the consideration of family affluence, males reported higher frequencies of each outcome than females. However, when assessing the relationship between family affluence and the risk of each outcome, it appears that risk estimates were higher in females than males for both outcomes, especially when examining the self-perceived affluence measurement. Furthermore, the associations within the female stratum remained significant when adjusted for additional confounders, while within the male stratum the associations were insignificant in addition to demonstrating a weaker than expected inverse relationship between affluence and both outcomes. A previous U.K. study by Nasr and colleagues contained similar results where they assessed this relationship, stratified the results by sex, and concluded that the risk estimates were higher in girls than boys [30].

While the results suggest that there is an inverse relationship between family affluence and fighting or fighting-related injury (which was exceptionally notable in the female population), the extent and direction of this relationship also depended on the affluence measurements in question. When using the FAS measurement, there were remarkable differences between the male and female adolescent population when assessing the association between family wealth and the outcomes of physical fighting and fighting-related injury. Within male adolescents, there was significant protective effect when comparing an individual’s risk of participating in a physical fight between the moderate and high material wealth groups, while the increased risk between the low and high affluence FAS groups was insignificant. While these findings are difficult to interpret without further qualitative or targeted quantitative investigation, we do recognize that for both the FAS and self-reported overall family affluence, there may be some misclassification if young people earn their own income, from part-time jobs for example, beyond their family situation. It is conceivable that engagement in part-time work would vary by age and also by level of family affluence. Engaging in part time work may also influence exposure to fighting since they may spend more of their time under supervised conditions where fighting is less likely to occur. More research needs to be done though to further understand this relationships.

Among the male adolescent population those in the lower FAS affluence group had significantly higher risk (almost 3 times) of obtaining a fighting-related injury than those in the high affluence group, whereas the decreased risk in the moderate affluence group was null when compared to the high affluence group. This appears to be a threshold effect where there is no significant difference between the high and moderate material wealth groups in regards to injury, but the risk sharply increases when comparing the low and high affluence groups. This may be due to a number of reasons. It is suspected that parents and adolescents from disadvantaged homes are not likely to be ‘reached’ by many health promotion resources, or parents in these areas may be unaware of the risks related to violence and are less exposed to interventions compared to parents from high or moderately affluent homes that have the minimum resources (such as electronics or transportation) that allow them to be ‘reached out’ [30]. It is also suspected that poorer families experience financial stress and may not have the time or resources to thoroughly supervise or monitor their children. While individuals in the low FAS group constitute a very small proportion of the HBSC sample (2.4 %), this cannot be ignored as this population contains a large percentage of the individuals who participate in physical fights and are injured as a result. It is important to address this issue due to detrimental health outcomes that result from physical fighting and fighting-related injury, and the mechanism behind this needs to be better understood. This threshold effect was not observed for the female population though and a socioeconomic gradient was observed instead. More research needs to be done to understand why this threshold effect was only witnessed in boys, and why being a boy of moderate affluence is a protective factor against fighting and injury.

When assessing area-level average household income, there was a small increase in the prevalence of physical fighting and fighting-related injury in the lower income group. When assessing its association with physical fighting, there was a significant increase in risk when comparing the lower affluence category to the higher affluence one, although this became insignificant for the multivariate model. This is suspected to be because of neighbourhood characteristics such as neighbourhood-level poverty and poorly maintained or unsafe residences that can weaken levels of social control and result in increased crime rates, which increases risk for violence and injuries [7, 21, 31].

The analyses with the area-level income measurement resulted in null findings when examining grade 9–10 students in the subset analysis. The area-level measurement may have yielded inconsistent results because the school postal code may be a poor approximation for area-level family income and there was likely insufficient power to detect injury effects as the estimated prevalence of fighting injuries was higher than the actual prevalence that was recorded. Future research may benefit from using a measure that more accurately estimates the affluence of an individual’s neighbourhood home rather than school as well as ensuring a large enough sample so as to have an adequate number of injury events occur.

Differences in risk suggest that the prevalence of physical fighting alone is higher in males than females, but the socioeconomic gradient in association with fighting and injury is stronger in females than males, where low income females are at exceptionally higher risk of obtaining both outcomes compared to higher income females. This suggests that when implementing public health interventions, focus on the male population at all affluence levels may be equally effective since it is suspected that male aggression and fighting is encouraged regardless of affluence level because of biological reasons such as increased testosterone levels, or social predispositions that reinforce gender norms [32, 33]. However when directing interventions at girls, it is imperative to focus public health efforts on low income females as they are at significantly higher risk of reporting both outcomes compared to females from highly affluent families. It is also important to involve parents, guardians and other grown up figures in a young person’s life who can influence and monitor their behaviour, especially aggressive ones [34]. Additional research can also be conducted to further examine why this socioeconomic gradient is much stronger in girls and contextually why girls from lower affluence families are more likely to report a physical fight or an injury related to fighting.

### Strengths and limitations

This study contains methodological strengths. For instance, it uses a large and nationally representative dataset that allows the results to be viewed with respect for the Canadian population. Further analysis focusing on a subset of grade 9–10 students accounted for additional variables not available in the entire dataset (such as the marijuana use variable). A comparison of the multiple measures of family wealth is another strength of this study since many measures exist and affluence is a construct that can be difficult to conceptualize and measure, especially in young people. This study explores various aspects of this construct and provides additional information for future research. This study also employs the use of robust error estimates for the regression analysis to account for the multi-level data.

There are also several methodological limitations in this study. Firstly, the area-level income variable used the school postal code to estimate area-level average household income, which may not be the most appropriate or accurate proxy since the school area may not be comparable to an individual’s neighbourhood. A more ideal method would be to use individual postal codes to approximate the wealth of an area that individuals reside in. Unfortunately in the 2009/2010 dataset there were significant amounts of missing data for home postal code and thus it was not an ideal measure. Secondly, the HBSC sample is nationally representative to Canada and it may be challenging to generalize these findings to different countries due to the underlying cultural differences in the acceptance of violence within different societies. There is potential for misclassification of the exposures and outcomes where the self-reported affluence question may misclassify participants depending on their frame of reference and perception of what “well off” truly means. The outcomes of fighting and fighting-related injury may also be misclassified if an injury occurred due to a fight during sports activities or martial arts and was classified as a sports-related injury instead. The injury survey items only asked about a participant’s “one most serious injury”. If there were multiple instances of injuries for an individual in the past year, then the true prevalence of fighting-related injuries may be under-estimated as some fighting injuries will be masked by more serious injuries caused by other circumstances. Material deprivation in the adolescent population also cannot be easily resolved since young people have little control over improving their family’s finances. This makes it a difficult point of intervention.

The FAS has been critiqued for its current validity since electronics and computers are becoming generally more affordable and may not be good affluence proxy measures. The FAS was updated for the 2013/2014 HBSC Study to accommodate these societal and economic changes. Although tested prior to inclusion in the HBSC international study, little information is available in regards to the validity and reliability of the self-perceived affluence and FAS variables specifically for Canadian students. Additionally, the family income at the area level did not have validity or reliability assessment for this study. This can be an important consideration for future research related to assessing socioeconomic indicators for Canadian children and adolescents.

## Conclusion

The present study indicates that a socioeconomic gradient exists where lower affluence is associated with a higher risk of participating in a physical fight or obtaining a fighting-related injury. Although the relationships stayed the same, this gradient varied in strength depending on the affluence measurement that was used to assess this relationship. Self-perceived affluence yielded the most significant results and showed a gradient effect; the FAS showed a significant threshold effect within males; and the area-level income showed a weaker gradient effect than the self-perceived affluence indicator and was only significant within female students. The variation in the results demonstrate that each affluence indicator may not measure affluence in the same way or to the same extent. Further analysis needs to be done to explore these measures and their underlying concepts and mechanisms. Further exploration of the interaction effect of sex in regards to the mechanism also needs to be better understood.

## Abbreviations

CI: confidence interval
FAS: family affluence scale
HBSC: health behaviour in school-aged children
RR: relative risk
SES: socioeconomic status
WHO: World Health Organization
